# ^1^H, ^13^C and ^15^N resonance assignments of the N-terminal intrinsically disordered region and WGR domain of human PARP2

**DOI:** 10.1007/s12104-026-10265-6

**Published:** 2026-04-09

**Authors:** Rajbinder K. Virk, Tae Hun Kim

**Affiliations:** 1 Department of Biochemistry, Case Western Reserve University, Cleveland, USA; 2 Case Comprehensive Cancer Center, Cleveland, USA

**Keywords:** Poly(ADP-ribose) polymerase 2, PARP2 N-terminal intrinsically disordered domain, PARP2 WGR domain, PARP2 DNA binding domain, Solution NMR, Backbone resonance assignments

## Abstract

Poly(ADP-ribose) polymerase 2 (PARP2) is a key sensor of DNA single-strand breaks that catalyzes ADP-ribosylation of itself and other substrates to initiate DNA repair. Human PARP2 contains an intrinsically disordered N-terminal domain (NTD) that mediates chromatin association and nuclear localization, a central WGR domain that recognizes DNA breaks, and a catalytic domain responsible for poly(ADP-ribose) synthesis. Despite its importance in genome maintenance and as a target of clinical PARP inhibitors, detailed information on the structural and dynamic properties of the NTD and WGR domains has remained limited. Here, we report the ^1^H, ^13^C, and ^15^N resonance assignments of the N-terminal intrinsically disordered region (residues 1–89) and the WGR domain (residues 90–212) of human PARP2. Resonance assignments were first obtained separately for each domain and subsequently transferred to a combined NTD–WGR construct. These assignments provide a foundation for future studies investigating the conformational dynamics, DNA recognition mechanisms, and allosteric regulation of PARP2 in chromatin and repair signaling.

## Biological context

Poly(ADP-ribose) polymerase 2 (PARP2) is a key enzyme in the base excision repair (BER) pathway that detects DNA single-strand breaks (SSBs) and catalyzes the synthesis of ADP-ribose polymers to recruit downstream repair factors (Amé et al. 1999; Ali et al. 2016; Chen et al. 2018; van Beek et al. 2021). Along with its paralog PARP1, PARP2 contributes to chromatin signaling during the DNA damage response and plays a critical role in maintaining genome stability (Amé et al. 2004; van Beek et al. 2021). Mutations or dysregulation of PARP2 have been implicated in cancer susceptibility and influence the cellular response to PARP inhibitors currently used in cancer therapy (Bryant et al. 2005; Murai et al. 2012; Ali et al. 2016).

Human PARP2 is composed of three major domains: an N-terminal intrinsically disordered domain (NTD) responsible for chromatin association and nuclear localization, a WGR (Trp-Gly-Arg) domain that mediates recognition of DNA breaks, and a C-terminal catalytic domain that carries out ADP-ribosylation (Meder et al. 2005; van Beek et al. 2021). The NTD contains a basic, low-complexity sequence that facilitates DNA-binding and has been shown to drive nuclear localization (Meder et al. 2005; Riccio et al. 2016). The WGR domain, in contrast, adopts a well-folded β-grasp topology that couples DNA binding to catalytic activation (Obaji et al. 2018). Together, these domains form a modular system that coordinates DNA damage recognition, chromatin engagement, and enzymatic activity (Bilokapic et al. 2020; Obaji et al. 2021; Smith-Pillet et al. 2025).

Despite its importance in genome maintenance and therapeutic targeting, detailed information on the structural and dynamic properties of the NTD and WGR domains has remained limited. Here, we report the ^1^H, ^13^C, and ^15^N backbone resonance assignments of the N-terminal intrinsically disordered region (residues 1–89) and the WGR domain (residues 90–212) of human PARP2. Assignments were first obtained separately for each domain and subsequently transferred to a combined NTD–WGR construct (1-212). These data provide the foundation for future investigations into the conformational dynamics, DNA recognition mechanisms, and allosteric regulation of PARP2 in chromatin and repair signaling.

## Methods and experiments

### Protein expression and purification

#### Cloning

The coding DNA sequences corresponding to the NTD, WGR, and NTD–WGR constructs were amplified by PCR using primers containing 5′ and 3′ overhangs complementary to the ends of the linearized pET-SUMO vector for Gibson assembly (NEB). Following amplification, PCR products were purified by gel extraction and inserted into the vector using Gibson assembly. The resulting plasmids were transformed into *E. coli*, and individual colonies were screened by Sanger sequencing to confirm the correct DNA sequences.

#### ^1^H, ^13^C, ^15^N labeled NTD (1–89)

The plasmid encoding residues 1–89 of human PARP2 (UniProt ID: Q9UGN5) was transformed into *E. coli* BL21(DE3) CodonPlus RIPL cells (Agilent) and plated on LB agar containing chloramphenicol and kanamycin. Following overnight incubation at 37 °C, colonies were selected and grown in 100 mL of LB medium with the same antibiotics for 16 h at 37 °C. The culture was centrifuged (3000 x g, 10 min, 23 °C), and the cell pellet was resuspended in 1 L of M9 minimal medium (6 g/L Na_2_HPO_4_, 3 g/L KH_2_PO_4_, 1 g/L NaCl, 1 g/L ^15^NH_4_Cl, pH 7.4) supplemented with 50 µg/mL kanamycin, 50 µg/mL chloramphenicol, 2 mM MgSO_4_, 0.1 mM CaCl_2_, 10 mg each of biotin and thiamine, and 4 g of ^13^C-glucose (Cambridge Isotope Laboratories). Cells were grown at 37 °C to an OD_600_ of 0.6, after which the temperature was reduced to 16 °C and incubation continued for 1 h prior to induction with 0.5 mM IPTG. Protein expression was carried out overnight at 16 °C. Following centrifugation (6000 x g, 30 min, 4 °C), the cell pellet was either stored at − 20 °C or processed immediately according to the following procedure.

The cell pellet was resuspended in lysis buffer (25 mM Tris-HCl, 500 mM NaCl, 20 mM imidazole, 2 mM β-mercaptoethanol, pH 8.0) supplemented with a protease inhibitor cocktail (0.5 mM PMSF, 1 mM benzamidine-HCl, 0.5 µg/mL of aprotinin, antipain, leupeptin and pepstatin A) and lysed by sonication on ice (2 s on/2 s off cycles, 10 min total, 40% amplitude). The lysate was cleared by centrifugation (20,000 × g, 30 min, 4 °C), and the supernatant was loaded onto Ni-NTA resin pre-equilibrated with lysis buffer. The column was washed with 20 column volumes of lysis buffer, and the bound His–SUMO–NTD protein was eluted using elution buffer (25 mM Tris, 500 mM NaCl, 300 mM imidazole, 2 mM β-mercaptoethanol, pH 8.0). Eluted fractions were analyzed by SDS–PAGE, and the His–SUMO tag was cleaved using in-house–prepared ULP1 protease during dialysis against buffer (25 mM HEPES, 50 mM NaCl, 2 mM β-mercaptoethanol, pH 7.4). After ULP1 digestion, the sample was reapplied to a second Ni–NTA column in the presence of 30 mM imidazole to remove the affinity tag. Following confirmation of tag removal by SDS–PAGE, the sample was further purified using a HiTrap SP HP cation-exchange column (Cytiva) equilibrated with buffer A (25 mM HEPES, 2 mM β-mercaptoethanol, pH 7.4). Elution was performed using buffer B (25 mM HEPES, 1 M NaCl, 2 mM β-mercaptoethanol, pH 7.4) under a linear gradient from 0% to 100% buffer B over 50 mL. Fractions containing the NTD were pooled, concentrated, and subjected to size-exclusion chromatography using a HiLoad 16/600 Superdex 75 pg column (Cytiva) equilibrated with gel filtration buffer (25 mM Tris, 150 mM NaCl, 2 mM β-mercaptoethanol, pH 7.4). The purified NTD was buffer-exchanged into NMR buffer (25 mM MES, 2 mM DTT, 5% (v/v) D_2_O, pH 5.5) for NMR spectroscopy. The final protein concentration used for NMR data acquisition was 1.0 mM.

#### ^1^H, ^13^C, ^15^N labeled WGR (90–212)

Procedures for expression and purification of uniformly ^1^H, ^13^C, ^15^N-labeled WGR (residues 90–212) were identical to those described for the NTD, except for the following modifications. Protein expression was induced at 20 °C instead of 16 °C. During purification, a HiTrap Heparin HP affinity column (Cytiva) was used in place of the HiTrap SP HP cation exchange column. The protein was buffer-exchanged into NMR buffer consisting of 25 mM MES, 4 mM TCEP and 5% (v/v) D_2_O (pH 5.5). The final protein concentration used for NMR data acquisition was 0.37 mM.

#### ^1^H, ^15^N labeled NTD-WGR (1-212)

Procedures for expression and purification of uniformly ^1^H, ^15^N-labeled NTD–WGR (residues 1–212) were identical to those used for the WGR construct, with the following modifications. Due to the low expression yield, a total culture volume of 8 L of M9 minimal medium supplemented with ^15^NH_4_Cl and unlabeled glucose was required to obtain a single NMR sample in 25 mM MES, 4 mM TCEP and 5% (v/v) D_2_O (pH 5.5). For resonance assignment, chemical shift information from the individually assigned NTD and WGR domains was used to aid assignment of the ^1^H – ^15^N HSQC spectrum of the NTD–WGR construct. The final protein concentration for NMR data acquisition was 0.73 mM.

#### NMR spectroscopy

NMR samples of uniformly ^1^H, ^13^C, and ^15^N-labeled PARP2 NTD, and WGR constructs were prepared in 25 mM MES (pH 5.5) containing 4 mM TCEP/ 2 mM DTT and 5% (v/v) D_2_O. Final protein concentrations ranged from 0.4 to 1.0 mM. Approximately 350 µL of each sample was loaded into a 5 mm Shigemi tube for data collection. NMR experiments were performed at 288.15 K on a Bruker Avance NEO 700 MHz spectrometer equipped with a TCI cryoprobe optimized for ^1^H detection and controlled by TopSpin 4.2 software. Sequential backbone assignments were achieved using a conventional suite of triple resonance experiments, including HNCA, HNCACB, CBCA(CO)NH, HN(CO)CA, HNCO, HAHB(CBCACO)NH and HNN for sequential ^1^H_N_–^15^ N correlations (Panchal et al. 2001) in combination with 2D ^1^H,^15^N HSQC spectra. For the NTD–WGR construct, resonance correlations were cross-referenced with the independently assigned spectra of the NTD and WGR domains to confirm chemical shift consistency. Backbone assignments of the isolated NTD were initially obtained from triple-resonance experiments recorded at 283.15 K. For comparison with the NTD–WGR construct, additional HNCO spectra were recorded at 288.15 K, and assignments were transferred accordingly. Chemical shift data recorded at both temperatures have been deposited in the BMRB under accession number 53520. All spectra were processed using NMRPipe v2024.061.14.29 (Delaglio et al. 1995) and analyzed with CcpNmr Analysis v3.3.4 (Skinner et al. 2016). Non-uniformly sampled (NUS) datasets were reconstructed using the SMILE algorithm within the NMRPipe processing suite. Secondary structure (SS) probabilities were obtained from backbone chemical shifts using TALOS-N (Shen and Bax 2013).

#### Extent of assignment and data deposition

**Fig. 1:**
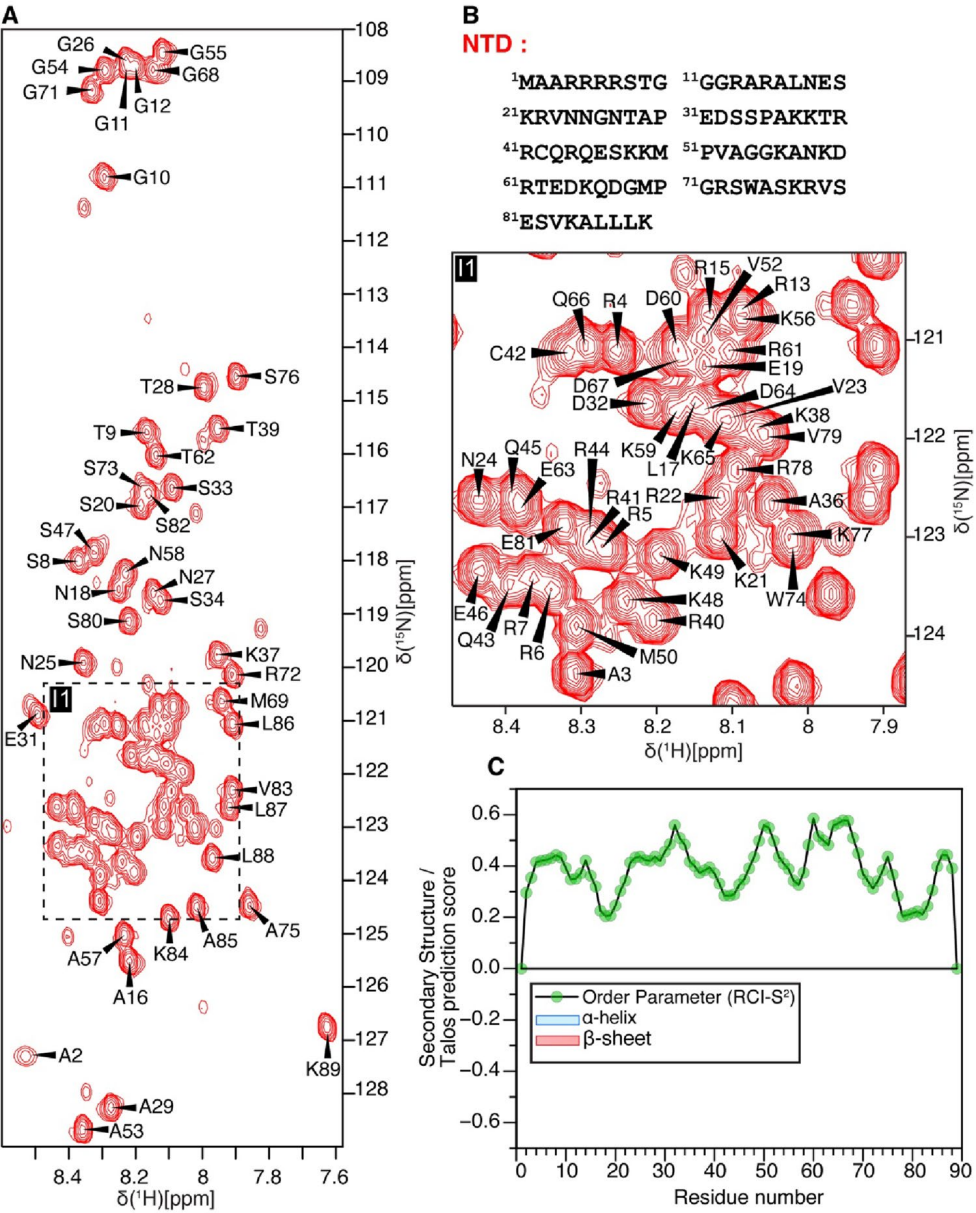
Backbone assignments of the N-terminal domain (NTD) of PARP2. (**A**) Two-dimensional ^1^H–^15^N projection of the three-dimensional HNCO spectrum of the NTD (BMRB accession number 53520) recorded at 283.15 K, with assigned backbone amide resonances labeled. (**B**) Amino acid sequence of the NTD (residues 1–89). (**C**) Residue-wise RCI-S^2^ order parameters predicted by TALOS-N from Cα, Cβ, C’, and HN chemical shifts. TALOS-N analysis indicates predominantly coil propensity with no α-helical or β-sheet secondary structure across the sequence. The relatively low RCI-S^2^ values (green scatter plot) suggest increased backbone flexibility, consistent with the intrinsic disorder nature of the NTD

**Fig. 2:**
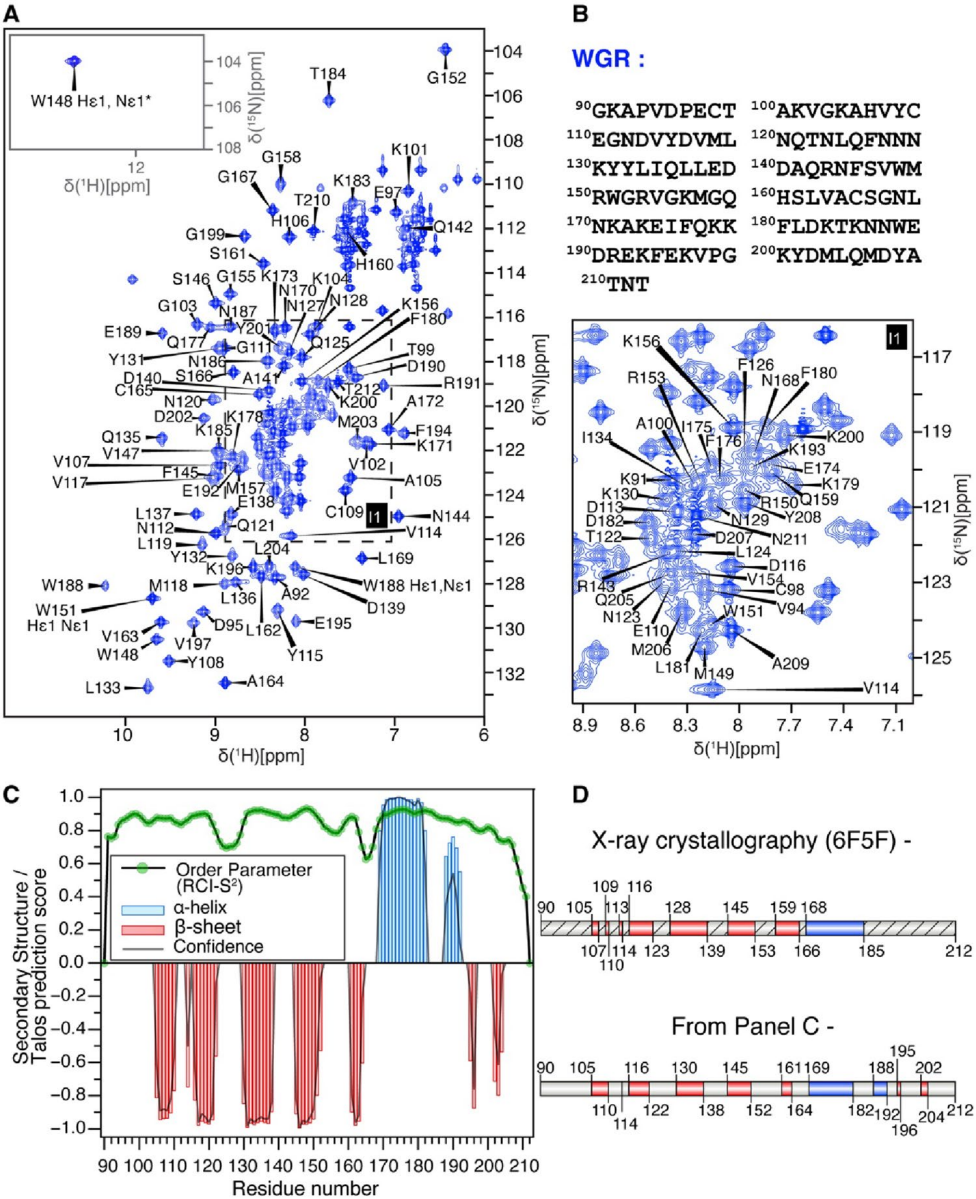
Backbone assignments of the WGR domain of PARP2. (**A**) Two-dimensional ^1^H–^15^N HSQC spectrum of the WGR domain (BMRB accession number 53521) recorded at 288.15 K, with assigned backbone amide resonances labeled. The resonance marked with an asterisk (*) is aliased in the displayed spectral window; its original position is at δ(¹⁵N) = 135 ppm. (**B**) Amino acid sequence of the WGR domain (residues 90–212). (**C**) Secondary structure probabilities derived from chemical shifts showing β-strands (red, plotted as negative values) and α-helices (blue); TALOS-N predicted RCI-S^2^ values are shown as green scatter points. (**D**) Secondary structure elements from the X-ray structure (PDB ID: 6F5F) derived using DSSP in PyMOL. The overall architecture agrees with the TALOS-N predictions

The ^1^H–^15^N HSQC spectra of the PARP2 NTD (residues 1–89, Fig. 1) and WGR (residues 90–212, Fig. 2) constructs displayed well-dispersed resonances, indicating monodisperse and properly folded states under the experimental conditions. Backbone resonance assignments for the NTD were obtained with substantial completeness, including 100% assignment of C′, Cα, and Cβ resonances. Assignment levels of 97.65% for HN and 94.32% for backbone ^15^N were achieved as determined using the assignment statistics module in CcpNmr Analysis. For the WGR domain, backbone assignments were also largely complete, with 100% assignment of C′ and 99.19% of Cα resonances. Assignment completeness was 99.17% for HN, 97.54% for ^15^N, 92.37% for Hα, 85.02% for Hβ and 93.04% for Cβ resonances. The indole N–H resonances of tryptophan side chains were assigned by site-directed mutagenesis, in which W148, W151, and W188 were individually mutated to tyrosine. Secondary structure predicted by TALOS-N for the NTD and WGR domains is shown in Figs. 1C and 2C, respectively. The NTD exhibits no significant secondary structure, consistent with its intrinsically disordered nature, and displays uniformly low RCI-S² values, indicative of high backbone dynamics. In contrast, the WGR domain exhibits strong β-strand and α-helical propensities, along with elevated TALOS-N predicted order parameters (RCI-S^2^ values ranging from 0.7 to 0.9), consistent with well-defined secondary structure elements and a rigid, folded architecture. Notably, although the crystal structure depicts residues 185–212 as largely unstructured, TALOS-N analysis of the NMR chemical shifts predicts some β-strand propensity in the C-terminal segment. Nevertheless, the TALOS-N secondary structure predictions for the WGR core domain overall are highly consistent with the X-ray crystal structure (Obaji et al. 2018). Secondary structure elements derived from the crystal structure were assigned using DSSP as implemented in PyMOL (Schrödinger, LLC). The domain architecture and corresponding secondary structure elements are shown in Fig. 2D; the representation was generated using IBM 2.0 (Liu et al. 2015).

**Fig. 3:**
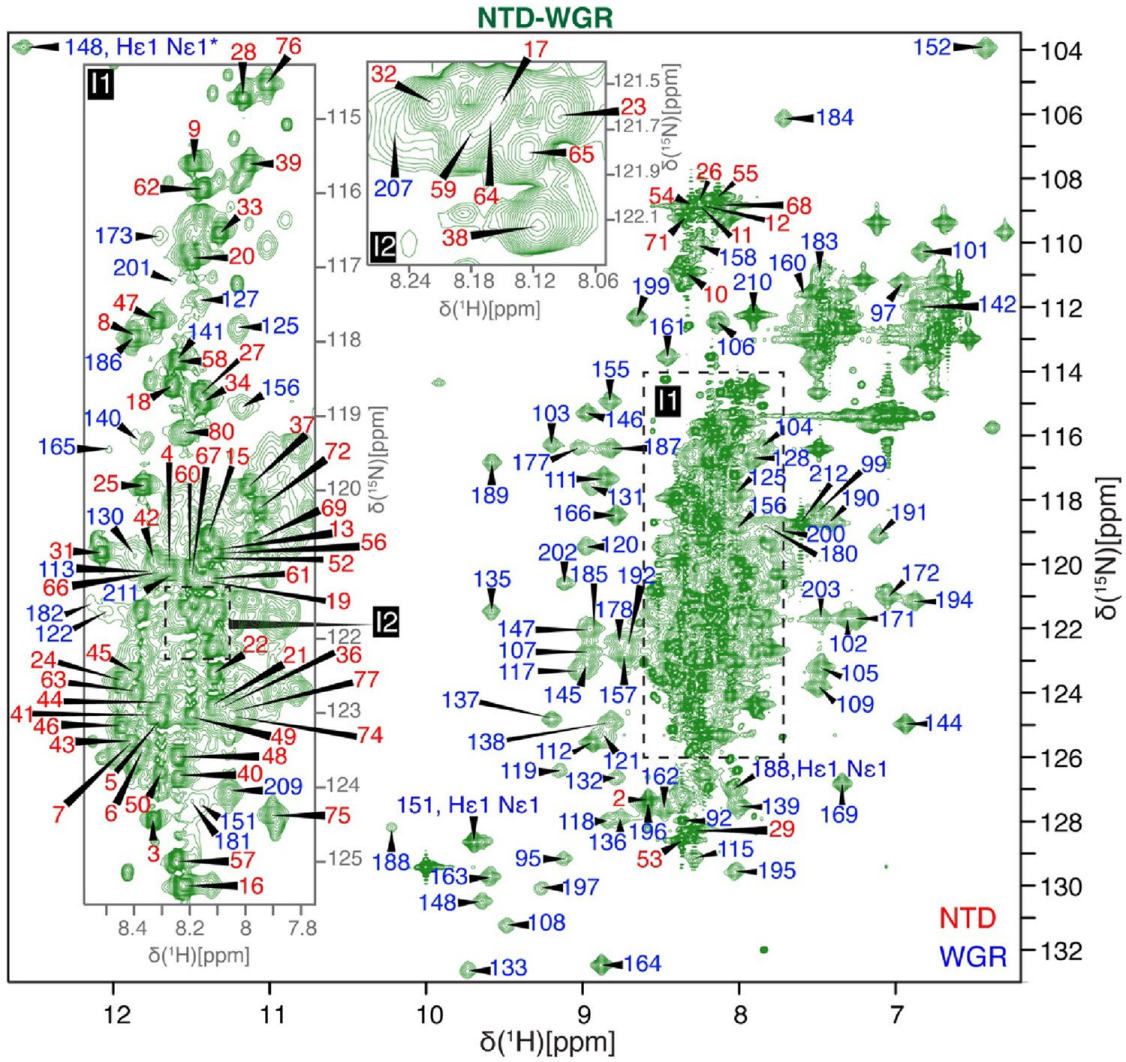
Two-dimensional ^1^H–^15^N HSQC spectrum of the NTD–WGR construct (residues 1-212) of PARP2 recorded at 288.15 K (BMRB accession number 53522). Resonances labeled in red correspond to the NTD, and those labeled in blue correspond to the WGR domain. Assignments were transferred from spectra of the individual domains recorded at 288.15 K to the NTD–WGR construct. The resonance marked with an asterisk (*) is aliased in the displayed spectral window; its original position is at δ(¹⁵N) = 135 ppm

Assignments for the NTD–WGR construct were guided by the individually assigned NTD and WGR spectra (Fig. 3). Due to the low expression yield, which required approximately 8 L of M9 minimal medium to obtain a single ^15^N-labeled sample at sufficient concentration for NMR experiments, only the ^1^H, ^15^N-labeled construct was used for NMR experiments. For the NTD–WGR construct, backbone amide assignments were obtained for 160 residues. After excluding proline residues, this corresponds to approximately 78% assignment completeness. The remaining 45 missing assignments are distributed between the domains, with 14 residues in the NTD (residues 1–89) and 31 residues in the WGR domain (residues 90–212). In the NTD, missing assignments cluster around residues 81–89, where peaks shift or broaden in the NTD–WGR spectrum. In the WGR domain, most missing assignments arise from spectral overlap and reduced peak intensity.

All assigned chemical shift data have been deposited in the Biological Magnetic Resonance Data Bank (BMRB) under accession numbers 53520 (NTD), 53521 (WGR), and 53522 (NTD–WGR).

## Data Availability

The chemical shift assignments obtained in this study are available in the Biological Magnetic Resonance Data Bank (BMRB, http://bmrb.io) under accession number 53520 (NTD), 53521 (WGR), and 53522 (NTD-WGR).
